# Tunable Doping of Rhenium and Vanadium into Transition Metal Dichalcogenides for Two‐Dimensional Electronics

**DOI:** 10.1002/advs.202004438

**Published:** 2021-04-02

**Authors:** Shisheng Li, Jinhua Hong, Bo Gao, Yung‐Chang Lin, Hong En Lim, Xueyi Lu, Jing Wu, Song Liu, Yoshitaka Tateyama, Yoshiki Sakuma, Kazuhito Tsukagoshi, Kazu Suenaga, Takaaki Taniguchi

**Affiliations:** ^1^ International Center for Young Scientists (ICYS) National Institute for Materials Science (NIMS) Tsukuba 305‐0044 Japan; ^2^ Nanomaterials Research Institute National Institute of Advanced Industrial Science and Technology AIST Central 5 Tsukuba 305‐8564 Japan; ^3^ Center for Green Research on Energy and Environmental Materials (GREEN) National Institute for Materials Science (NIMS) Tsukuba 305‐0044 Japan; ^4^ International Center for Materials Nanoarchitectonics (WPI‐MANA) National Institute for Materials Science (NIMS) Tsukuba 305‐0044 Japan; ^5^ Department of Physics Tokyo Metropolitan University Hachioji 192‐0397 Japan; ^6^ Institute of Materials Research and Engineering Agency for Science Technology and Research Singapore 138634 Singapore; ^7^ Institute of Chemical Biology and Nanomedicine (ICBN) College of Chemistry and Chemical Engineering Hunan University Changsha 410082 P. R. China; ^8^ Research Center for Functional Materials National Institute for Materials Science (NIMS) Tsukuba 305‐0044 Japan

**Keywords:** chemical vapor deposition, contact, doping, electronics, salt

## Abstract

Two‐dimensional (2D) transition metal dichalcogenides (TMDCs) with unique electrical properties are fascinating materials used for future electronics. However, the strong Fermi level pinning effect at the interface of TMDCs and metal electrodes always leads to high contact resistance, which seriously hinders their application in 2D electronics. One effective way to overcome this is to use metallic TMDCs or transferred metal electrodes as van der Waals (vdW) contacts. Alternatively, using highly conductive doped TMDCs will have a profound impact on the contact engineering of 2D electronics. Here, a novel chemical vapor deposition (CVD) using mixed molten salts is established for vapor–liquid–solid growth of high‐quality rhenium (Re) and vanadium (V) doped TMDC monolayers with high controllability and reproducibility. A tunable semiconductor to metal transition is observed in the Re‐ and V‐doped TMDCs. Electrical conductivity increases up to a factor of 10^8^ in the degenerate V‐doped WS_2_ and WSe_2_. Using V‐doped WSe_2_ as vdW contact, the on‐state current and on/off ratio of WSe_2_‐based field‐effect transistors have been substantially improved (from ≈10^–8^ to 10^–5^ A; ≈10^4^ to 10^8^), compared to metal contacts. Future studies on lateral contacts and interconnects using doped TMDCs will pave the way for 2D integrated circuits and flexible electronics.

## Graphical abstract

A chemical vapor deposition method using mixed molten salts is established for vapor–liquid–solid growth of Re‐ and V‐doped transition metal dichalcogenide (TMDC) monolayers. Tunable semiconductor to metal transition is observed in the as‐grown Re‐ and V‐doped TMDC monolayers. Using heavily V‐doped WSe_2_ as van der Waals contact, the performance of WSe_2_‐based field‐effect transistors is improved by 1–3 orders of magnitude compared to Au and Pd.

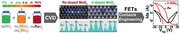

Two‐dimensional (2D) materials, e.g., graphene, h‐BN, and transition metal dichalcogenides (TMDCs), are important building blocks for future electronics and optoelectronics. Among them, the group VI semiconducting TMDCs are potential candidates for the channel materials of field‐effect transistors (FETs) as they exhibit reasonable carrier mobility (tens to hundreds cm^2^ per Vs) and high current on/off ratio (up to 10^8^).^[^ ^1^, ^2^, ^3^, ^4^ ^]^ Furthermore, they are endowed with mechanical flexibility and high optical transparency for applications in next‐generation, light‐weight, flexible, and wearable electronics.^[^ ^5^ ^]^ However, one serious issue regarding 2D TMDC‐based electronics is to overcome the strong Fermi level pinning effect that fixes Schottky barrier heights (SBHs) at the TMDC/metal contact interface.^[^ ^6^, ^7^, ^8^, ^9^, ^10^, ^11^, ^12^ ^]^ It hinders carrier modulation, causing high contact resistance in the TMDC‐based devices as a consequence. To solve this problem, numerous efforts have been devoted to the contact engineering. For instance, vertical or lateral contact has been established with metallic 2D materials.^[^ ^13^, ^14^, ^15^, ^16^, ^17^, ^18^, ^19^, ^20^, ^21^, ^22^, ^23^, ^24^ ^]^ Besides, instead of direct metal deposition, prepatterned metal electrodes are transferred as contacts to avoid damages or strain to the 2D TMDCs, which cause high contact resistance.^[^ ^25^ ^]^ Meanwhile, ion implantation is a well‐established doping technique to modulate the carrier density and to realize ohmic contact in silicon‐based devices. Similar, substitutional doping of 2D TMDCs provides a brand‐new strategy for the contact engineering and carrier modulation of 2D TMDC‐based electronics.^[^ ^26^, ^27^ ^]^

In the last 5 years, many important advances in TMDC doping have been achieved by chemical vapor deposition (CVD) method. These include direct growth with a mixture of transition metal oxides/halides,^[^ ^26^, ^28^, ^29^, ^30^, ^31^ ^]^ liquid‐phase precursors,^[^ ^32^, ^33^, ^34^ ^]^ or metal organic reactants (MOCVD),^[^ ^35^ ^]^ which remarkably enhance the electrical, magnetic, and catalytic properties of doped TMDCs. However, vaporization of the transition metal precursors from solid powders oftentimes leads to poor uniformity and controllability of as‐grown doped TMDCs. Furthermore, the high concentration of transition metal precursors and byproducts has also caused severe contaminations. Although the MOCVD shows good uniformity and controllability, as‐grown doped TMDCs still suffer from small grain size (sub‐micrometer level), not to mention the high cost involved. Therefore, a new synthetic technique compatible with conventional ambient‐pressure CVD, which can be handled with great feasibility and easy controllability, is highly demanding to precisely tune the doping and electrical properties of as‐grown TMDCs. Only thus can doped TMDCs make substantial contribution to future 2D electronics.

In this communication, we report a versatile, highly reproducible vapor–liquid–solid (VLS) growth of Re‐ and V‐doped TMDC monolayers using the molten salts, Na_2_MoO_4_, Na_2_WO_4_, NaReO_4_, and NaVO_3_. By adjusting the NaReO_4_ and NaVO_3_ ratio in their mixtures with Na_2_MoO_4_ or Na_2_WO_4_, high‐quality Re‐ and V‐doped TMDC (MoS_2_, WS_2_ and WSe_2_) monolayers with tunable composition can be easily grown by sulfurization and selenization. With the use of molten salts, the mixed salts are in liquid state during the TMDC growth. The mixture is thus has a higher spatial and composition uniformity compared to the vaporized transiton metal precursors. Besides, it has minimal contamination to the reaction chamber. Repeated growth of more than a hundred batches can be conducted using a single growth chamber under ambient pressure CVD process. We attribute this to the thin‐layer, spin‐coated molten salts used for growing Re‐, and V‐doped TMDCs. This is almost impossible for the doped TMDCs grown with vaporized precursors. In addition, the as‐grown Re‐ and V‐doped TMDCs can easily achieve a very large domian size up to 100 µm. Our spectroscopic studies using Raman and photoluminescence (PL) along with the scanning transmission electron microscopy (STEM) observations confirm the successful doping of Re and V in the as‐grown samples. The electrical properties of these Re‐ and V‐doped TMDC‐based FETs show possible carrier modulation. Distinct semiconductor to metal transition is observed with the increase of Re and V concentration. Highly conductive V‐doped WSe_2_ were employed as vertical vdW contact for WSe_2_‐FETs. Compared to typical metal contacts using Au and Pd, the on‐state current and on/off ratio of WSe_2_‐based FETs have been substantially improved from ≈10^–6^–10^–8^ A to 10^–5^ A and from ≈10^4^ to 10^8^, respectively. Our results demonstrate the great potentials of Re‐ and V‐doped TMDCs for future 2D TMDC‐based electronics.

To modify the electrical properties of group VI TMDCs, one effective way is via substitutional replacement of Mo and W elements with neighboring Re (electron donor), V, or Nb (electron acceptor) in periodic table (**Figure** **1**a). The band structure, carrier type, and electrical properties of 2D TMDCs can be systematically tuned with the dopant concentration. Our recent achievements in molten‐salt CVD, VLS growth of TMDCs show great promise for synthesizing doped TMDCs with high controllability and great feasibility.^[^ ^36^, ^37^ ^]^ Molten slats, e.g., Na_2_MoO_4_ and Na_2_WO_4_ have melting points of 687 °C and 698 °C, respectively. They are in liquid (molten) state because of their low vapor pressures at the typical growth temperatures of TMDC monolayers, around 750 °C. In stark contrast to the volatile transition metal oxides, chlorides, and oxychlorides, which grow TMDC monolayers in a vapor–solid–solid (VSS) mechanism,^[^ ^4^, ^38^ ^]^ these molten salts grow TMDC monolayers in a VLS mechanism.^[^ ^36^, ^37^ ^]^ In principle, mixed molten salts are expected to have better controllability than the vapor precursors in growing doped TMDCs. Here, we have chosen similar molten salts, NaReO_4_ (*T*_m_ = 414 °C) and NaVO_3_ (*T*_m_ = 630 °C) to grow the Re‐ and V‐doped TMDC monolayers, respectively. First, 20 × 10^−3^ m Na_2_MoO_4_, Na_2_WO_4_, NaReO_4_, and NaVO_3_ aqueous solution were prepared as source precursors. Then, the mixed salt solutions with different NaReO_4_ and NaVO_3_ ratios were prepared as illustrated in Figure 1b. Here and thereafter, *X* denotes the mixed salts of Na_2_MoO_4_‐NaReO_4_ (*X_ReMo_*), Na_2_MoO_4_‐NaVO_3_ (*X_VMo_*), Na_2_WO_4_‐NaReO_4_ (*X_ReW_*), and Na_2_WO_4_‐NaVO_3_ (*X_VW_*). *X^Re^* and *X^V^* represent the ratio of NaReO_4_ and NaVO_3_ in mixed salts, respectively. Later, the mixed salt solutions were spin‐coated onto sapphire substrates. Finally, Re‐ and V‐doped TMDC monolayers were grown by either sulfurizing or selenizing the mixed salts in an ambient‐pressure thermal CVD system (Figure 1c).

**Figure 1:**
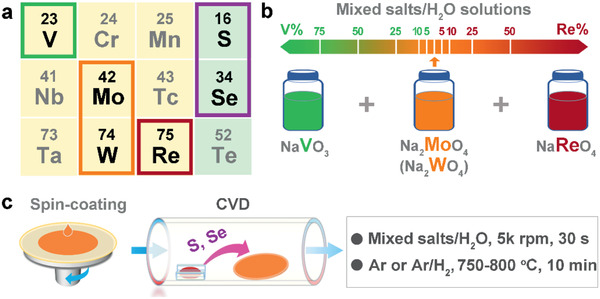
Strategy for the chemical vapor deposition (CVD) growth of two‐dimensional (2D) Re‐ and V‐doped transition metal dichalcogenides (TMDCs). a) A partial periodic table indicating the substitutional Re (electron donor) and V (electron acceptor) doping of 2D TMDCs (*M*S_2_, and *M*Se_2_, *M* = Mo, W). b) Mixed salt solutions with different NaReO_4_ and NaVO_3_ ratios are prepared as source precursors. c) Schematic illustrations of the spin‐coating of mixed salt solution onto growth substrate and the conditions employed in the CVD growth of Re‐ and V‐doped TMDC monolayers.

To investigate the growth of tunable Re‐ and V‐doped TMDC monolayers, the Na_2_MoO_4_‐NaReO_4_ (*X_ReMo_*) and Na_2_MoO_4_‐NaVO_3_ (*X_VMo_*) mixed salts were first used for the CVD growth of Re‐ and V‐doped MoS_2_ monolayers, respectively. Our X‐ray photoelectron spectroscopy (XPS) results demonstrate successful Re doping in the as‐grown MoS_2_ monolayers (Figure S1, Supporting Information). The optical images of the as‐grown Re‐ and V‐doped MoS_2_ monolayers are shown in Figure S2 (Supporting Information). Overall, the grain size and surface coverage of the as‐grown Re‐ and V‐doped MoS_2_ monolayers show an obvious decrease with increasing ratios of NaReO_4_ and NaVO_3_ in the mixed salts. For the Re‐doped MoS_2_ monolayers, a drastic decrease of grain size is observed, from ≈100, ≈20 to <10 µm with the $X_{ReMo}^{Re}$ increases from 5 to 10% and >25%, respectively. In contrast, the grain size of V‐doped MoS_2_ monolayers shows less sensitivity. Large grains of ≈100 µm in size can be grown even at a high $X_{VMo}^{V}$ of 50%. **Figure** **2**a shows a group photo of the Re‐ and V‐doped MoS_2_ monolayers transferred onto double‐side polished sapphire substrates. A gradual color change from light yellowish green (MoS_2_ monolayers) to light brown color is noted when the $X_{ReMo}^{Re}$ changes from 0 to 50%. It matches well with the absorption spectra, where both A‐ and B‐exciton peaks are gradually decreasing and disappeared at 50% $X_{ReMo}^{Re}$ (Figure 2b). As a result, the photoemission peaks from A excitons are also decreased and red‐shifted in the PL spectra (Figure 2c). This is because the substitutional Re doping changes the band structure of MoS_2_ monolayers. New defect states are introduced near the conduction band minimum.^[^ ^29^, ^39^ ^]^ Similarly, a gradual color change is observed in the V‐doped MoS_2_ monolayers (Figure 2a). The A and B‐excitons absorption peaks have totally diminished in the V‐doped MoS_2_ monolayers that were grown with 25% and higher $X_{VMo}^{V}$. More interestingly, a strong emission peak at lower energy of ≈1.63 eV with a broad FWHM of ≈200 meV (Figure 2c) is observed in the V‐doped MoS_2_ monolayers grown with 5%$\;X_{VMo}^{V}$. We attribute this new peak to the defect states above the valence band maximum caused by V doping into the lattice of MoS_2_ monolayers.^[^ ^40^, ^41^ ^]^ However, when the $X_{VMo}^{V}$ increases to 10%, only a very weak A‐exciton emission and a broad low‐energy defect‐related emission are observed. The PL is fully quenched beyond 25% $X_{VMo}^{V}$, suggesting a heavily doped degenerate state is achieved.

**Figure 2:**
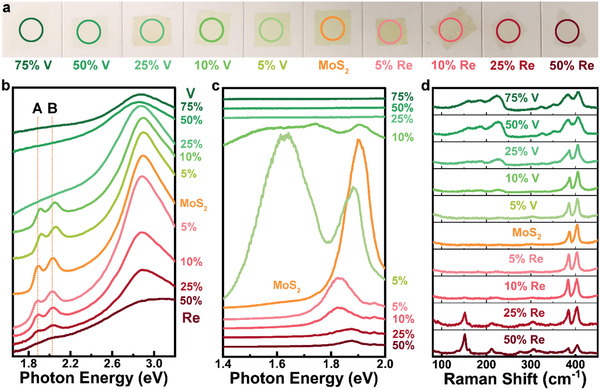
Spectroscopic characterization of Re‐ and V‐doped MoS_2_ monolayers. a) Optical images of the transferred Re‐ and V‐doped MoS_2_ monolayers on double‐side polished sapphire substrates. The circles indicate the area inspected in UV–Vis spectroscopy. b) Absorption spectra of the transferred Re‐ and V‐doped MoS_2_ monolayers shown in (a). c,d) Typical c) photoluminescence (PL) and d) Raman spectra of the Re‐ and V‐doped MoS_2_ monolayers transferred on SiO_2_/Si substrates. All the Re% and V% labeled in a–d) represent the NaReO_4_ and NaVO_3_ ratios in the mixed salt solutions, not the actual Re and V concentrations in the doped MoS_2_ monolayers. b–d) *y* axis of the plots represent intensity with arbitrary unit.

Figure 2d demonstrates the evolution of Raman spectra for the Re‐ and V‐doped MoS_2_ monolayers. The intrinsic MoS_2_ monolayers have two characteristic Raman modes, namely E_2g_^1^ at ≈386 cm^−1^ and A_1g_ at ≈404 cm^−1^ with a peak separation of ≈18 cm^−1^.^[^ ^42^ ^]^ For the Re‐doped MoS_2_ monolayers, new Raman modes recorded at 150, 211, 276, and 305 cm^−1^ are ascribed to the ReS_2_ monolayers.^[^ ^43^ ^]^ These new Raman modes indicate the formation of ReS_2_ domains in the Re‐doped MoS_2_ monolayers grown with ≥25% $X_{ReMo}^{Re}$. Whereas for the V‐doped MoS_2_ monolayers, new Raman modes appeared at 158, 187, 227, 323, and 351 cm^−1^ indicate the successful substitutional V doping in the MoS_2_ lattice.^[^ ^41^ ^]^

Similarly, we also conducted the CVD growth of Re‐ and V‐doped WS_2_ and WSe_2_ monolayers. Their optical images are shown in Figure S3 (Supporting Information). Their Raman spectra show solid evidence for the successful growth of Re‐ and V‐doped WS_2_ and WSe_2_ monolayers. New Raman modes and quenched PL are observed in these samples (Figure S4, Supporting Information). However, it is hard to grow Re‐doped WS_2_ and WSe_2_ monolayers due to the large formation energy.^[^ ^44^ ^]^ In the future, more efforts should be given to the growth of n‐type Re‐doped TMDC monolayers.^[^ ^45^ ^]^

The atomic structures of Re‐ and V‐doped TMDC monolayers were investigated by atomic‐resolution STEM. **Figure** **3**a–d shows four typical annular dark field (ADF) STEM images of Re‐ and V‐doped TMDC monolayers: Re‐doped MoS_2_, V‐doped MoS_2_, V‐doped WS_2_, and V‐doped WSe_2_, respectively. As shown in Figure 3a, Re atoms show brighter contrast than the Mo atoms since the ADF contrast is propotional to *Z* ^2^ (Re (*Z* = 75), Mo (42), S (16)). In most area of the Re‐doped MoS_2_ monolayers (grown with 25% $X_{ReMo}^{Re}$), the Re dopants are uniformly dispersed in the MoS_2_ lattice with a concentration of ≈2.1%, which is probably limited by the solubility. On the other hand, we have also found small ReS_2_ domains containing Mo dopants, in a size of ≈150 × 150 nm^2^, embeded in the host MoS_2_ (Figure S5, Supporting Information). Note that the ReS_2_ presents distorted 1T phase. Such phase separation is more pronounced at higher Re doping concentration when the $X_{ReMo}^{Re}$ exceeds 25%. This matches well with the observation of enhanced ReS_2_ Raman modes in the Re‐doped MoS_2_ monolayers grown with 25% and 50% $X_{ReMo}^{Re}$ (Figure 2d).

**Figure 3:**
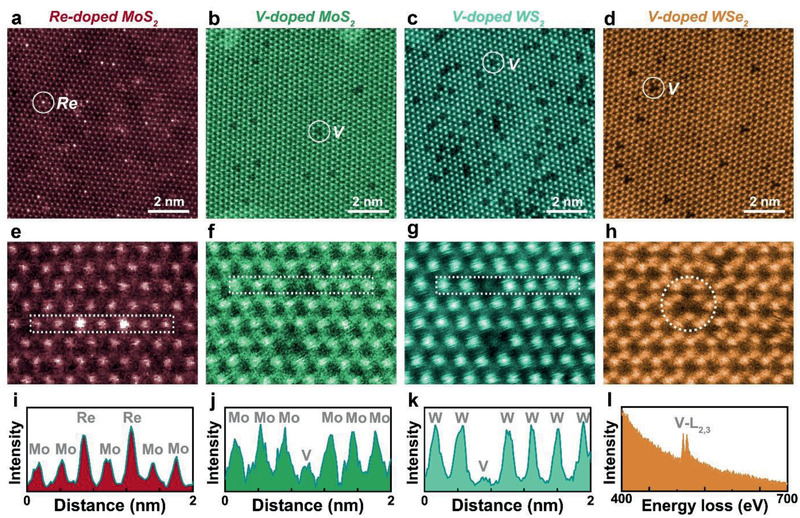
Atomic structures of Re‐ and V‐doped transition metal dichalcogenide (TMDC) monolayers. a–d) Low magnification annular dark field (ADF) scanning transmission electron microscopy (STEM) images of a) Re‐doped MoS_2_ (25% $X_{ReMo}^{Re}$), b) V‐doped MoS_2_ (25% $X_{VMo}^{V}$), c) V‐doped WS_2_ (5% $X_{VW}^{V}$), and d) V‐doped WS_2_ (5% $X_{VW}^{V}$), respectively. e–h) The corresponding high magnification ADF–STEM images. i–k) The ADF intensity profiles of Re dopants in MoS_2_, V dopants in MoS_2_ and WS_2_ extracted from the dotted boxes in e–g) identifying the position of Re and V from the intensity. l) The EELS spectrum of V dopant in WSe_2_ taken from the dotted circle in h).

Figure 3b‐d shows the ADF‐STEM images of V‐doped MoS_2_, WS_2_, and WSe_2_ monolayers, respectively. The V dopants are uniformly dispersed in these samples without phase segregation, indicating a good miscibility of V atoms in these TMDC monolayers.^[^ ^34^, ^35^, ^46^ ^]^ From the STEM images, we estimate ≈2.9%, ≈4.7%, and ≈2.7% of V atoms are doped in 25% $X_{VMo}^{V}$‐MoS_2_, 5% $X_{VW}^{V}$‐WS_2_, and 5% $X_{VW}^{V}$‐WSe_2_ monolayers, respectively. Compared to the W‐based TMDCs, the doping concentration of V in MoS_2_ monolayers is not as high (or merely equivalent) even with an increased amount of NaVO_3_ (25%) used. The result implies a higher compability of V in WS_2_ than WSe_2_ over MoS_2_.

Substitutional replacement of Mo or W with electron donor (Re) and acceptor (V) can dramatically change the electronic structure, carrier types, and conductivity of TMDC monolayers. **Figure** **4**a demonstrates the typical FET transport curves of Re‐ and V‐doped MoS_2_ monolayers. With increasing NaReO_4_ ratio in the mixed salts, we see a dramatic shift of threshold voltage to more negative gate bias, indicating a strong electron doping in the as‐grown Re‐doped MoS_2_ monolayers. Compared to the intrinsic MoS_2_ monolayers, the on‐state current and current on/off ratio decreased by two orders of magnitude (from ≈10^–5^ to 10^–7^ A; 10^8^ to 10^6^) for 5% $X_{ReMo}^{Re}$‐MoS_2_ monolayers, which mainly due to the scattering caused by the Re atoms in MoS_2_ lattice. Degenerate electron transport and improved conductivity are achieved in 10% and 25% $X_{ReMo}^{Re}$‐MoS_2_ monolayers. For the V‐doped MoS_2_ monolayers, the on‐state current for electron transport shows a steady decrease but still maintain a high current on/off ratio up to ≈10^7^ for 5% and 10% $X_{VMo}^{V}$‐MoS_2_ monolayers. Meanwhile, the threshold voltage shows a positive shift due to the strong hole doping. A degenerate doping is achieved in 25–75% $X_{VMo}^{V}$‐MoS_2_ monolayers, poor gate‐tunability, and metallic transport behavior are observed in these samples.

**Figure 4:**
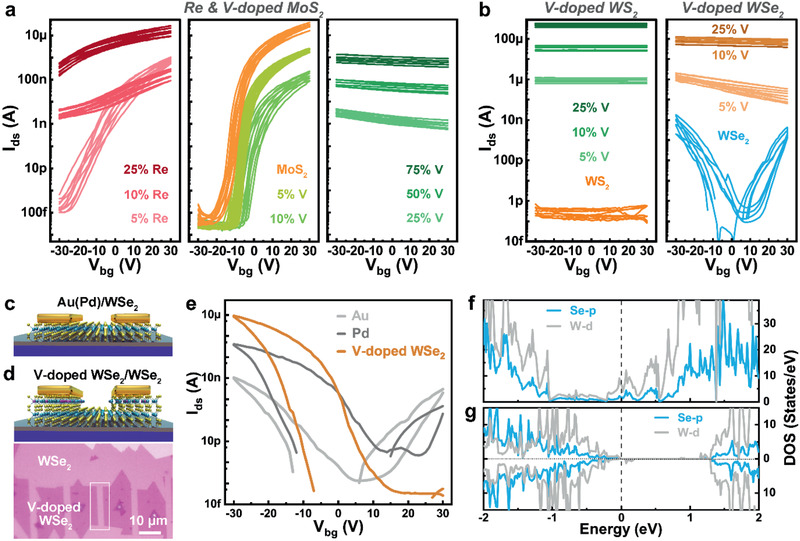
Tunable electrical properties of Re‐ and V‐doped transition metal dichalcogenides (TMDCs) and contact engineering of WSe_2_ field effect transistors (FETs). a) Typical transport curves of Re‐ and V‐doped MoS_2_ monolayers. b) Typical transport curves of V‐doped WS_2_ and WSe_2_ monolayers. All the Re% and V% represent the NaReO_4_ and NaVO_3_ ratios in the mixed salt solutions. c) Schematic of Au or Pd contact for WSe_2_‐FETs. d) Schematic and optical image of V‐doped WSe_2_ as vdW contact for a WSe_2_‐FET. e) Comparison of transport properties of WSe_2_‐FETs with three kinds of contacts: Au, Pd, and V‐doped WSe_2_. f,g) Projected density of states (PDOS) of WSe_2_ monolayers contacting with f) Au and g) V‐doped WSe_2_, respectively.

Figure 4b shows the typical FET transport curves of V‐doped WS_2_ and WSe_2_ monolayers. Intrinsic WS_2_ and WSe_2_ monolayers show poor transport properties when Au was used as contacts. With increasing ratios of NaVO_3_, a steady increment of hole conductivity is achieved in the V‐doped samples. The conductivity in 10–25% $X_{VW}^{V}$‐WS_2_ (WSe_2_) monolayers show a dramatic increase up to 10^8^ times, compared to the intrinsic WS_2_ and WSe_2_ at zero gate bias. This matches well with the results of density functional theory (DFT) calculations. With the increase of the doping concentration of V atoms in WSe_2_ monolayers, a steady down‐shift of the Fermi level into the valence band is observed (Figure S6, Supporting Information), indicating the increase of hole concentration. The calculated partial charge density associated with states near the Fermi level in 11.1% V‐doped WSe_2_ (Figure S6e, Supporting Information) is quite delocalized compared to that in 1% V‐doped WSe_2_ (Figure S6c, Supporting Information), which localized around the dopants. This indicates a higher hole conductivity in the 11.1% V‐doped WSe_2_. In addition, much higher hole conductivity is achieved in the V‐doped WS_2_ and WSe_2_ than V‐doped MoS_2_. We attribute this to the feasible high concentration of ionized V dopants in WSe_2_ and WS_2_ monolayers (Figure 3c–d and Figure S6, Supporting Information). Meanwhile, high ratio of NaVO_3_ (25% $X_{VW}^{V}$) leads to the growth of multilayer V‐doped WS_2_ and highly reactive V‐doped WSe_2_ monolayers. The 25% $X_{VW}^{V}$‐WSe_2_ monolayers generate holes soon after exposed to the ambient atmosphere, which accounts for the similar electrical properties observed in the 25% and 10% $X_{VW}^{V}$‐WSe_2_ monolayers.

For the contact engineering of 2D electronics, the metallic 10% $X_{VW}^{V}$‐WSe_2_ monolayers were employed as vdW contacts for WSe_2_ FETs due to their high conductivity and stability. Figure 4c–d illustrates two types of contacts for WSe_2_‐FETs: one is the widely used Au or Pd metal contact (Figure 4c). The other is the degenerately V‐doped WSe_2_ contact. An optical image showing the vdW contacts formed by transferring the etched V‐doped WSe_2_ monolayers onto WSe_2_ monolayers is depicted in Figure 4d. Detailed fabrication process is presented in Figure S7 (Supporting Information) and Experimental Section. Figure 4e is the typical transport curves of WSe_2_‐FETs with three different contacts, Au‐, Pd‐, and V‐doped WSe_2_. When Au was employed as contact, the WSe_2_‐FETs show an obvious ambipolar transport behavior with on‐state current of ≈10 nA and a current on/off ratio of ≈10^4^. Pd is ideal for hole transport in WSe_2_‐FETs due to its high work function. Changing the contact metal from Au to Pd has improved the on‐state current for hole transport by two orders of magnitude (from ≈10^–8^ to 10^–6^ A). Meanwhile, when V‐doped WSe_2_ was used as contacts, the on‐state current for hole transport is enhanced by three orders of magnitude compared to Au (from ≈10^–8^ to 10^–5^ A), also showing a much better result than the Pd contact. This enables an improved current on/off ratio of V‐doped WSe_2_ contacted WSe_2_‐FETs, reaching a high value of 10^8^. Furthermore, compared to the metal contacts, the electron transport can be fully quenched at positive gate bias in V‐doped WSe_2_ contact. This implies a promising application in low‐power consumption nanoelectronics.

To better understand the difference between metal (Au) and V‐doped WSe_2_ vdW contact for WSe_2_‐FETs, corresponding contact geometries were built and simulated using the first‐principles DFT calculations (Figure S8, Supporting Information). For the Au‐WSe_2_ contact, the calculated projected density of states (PDOS) indicates that Au can metallize WSe_2_ monolayer strongly and fill the bandgap with states (Figure 4f). This further confirms that the metal‐induced gap states (MIGS) are formed in the contact between traditional metals and TMDC monolayers. The MIGS and large strain at the interface result in the high contact resistance.^[^ ^6^, ^7^, ^8^, ^9^, ^10^, ^11^, ^12^ ^]^ Therefore, instead of the conventional metal deposition process, Au eletrodes were transferred as 3D vdW contacts in the TMDC‐based electronic devices to elimate the MIGS and large strains generated.^[^ ^25^ ^]^ In stark contrast to the Au contact, the the WSe_2_‐FETs show p‐type behavior with the V‐doped WSe_2_ vdW contact. The smaller metallization effect preserves the intrinsic bandgap of WSe_2_ monolayer (Figure 4g and Figure S6b, Supporting Information). Our simulation results well explained the experimental observations, suggesting the V‐doped WSe_2_ monolayers as promising electrode material for the p‐type WSe_2_‐FETs. In addition, because of the similar lattice parameters, the negligible distortion/strain at junction of the intrinisic and V‐doped WSe_2_ monolayers has contributed to the low‐resistance contacts.^[^ ^12^ ^]^

In summary, CVD method using mixed molten salts is highly promising for the VLS growth of Re‐ and V‐doped TMDC monolayers. Tunable composition, optical, and electrical properties are achieved in the Re‐ and V‐doped TMDC monolayers. The metallic V‐doped WSe_2_ monolayers are ideal p‐type vdW contact for the WSe_2_‐FETs. Much improved device performance is observed compared to the traditional Au and Pd contacts. Meanwhile, in order to obtain a good contact for electron transport, more efforts should be devoted to the growth of degenerate Re‐doped TMDCs or explore new low‐work‐function 2D metals. These Re‐ and V‐doped TMDCs are expected to bring profound impacts to the 2D TMDC‐based electronics as promising electrodes and interconnects. In addition, as 2D lateral contacts and PN junctions can also be created using patterned growth with molten slats, this will further improve the incorporation and functionality of 2D TMDC‐based integrated circuits.

## Experimental Section

##### Preparation of Mixed Salt Precursors

First, 20 × 10^−3^ m Na_2_MoO_4_ (99%, Sterm Chemicals), Na_2_WO_4_ (99+%, Strem Chemicals), NaReO_4_ (99.95%, Alfa Aesar), and NaVO_3_ (96%, Alfa Aesar) aqueous solutions were prepared by dissolving the salts in DI water, respectively. Then, the aqueous salt solutions were mixed in designated ratios for growing Re‐ and V‐doped TMDC monolayers. One‐side polished sapphire substrates (c‐plane, Shinkosha) were treated with UV‐O_3_ for 30 min to obtain hydrophilic surface. The mixed salt solutions were then spin‐coated on the treated sapphire substrates with a speed of 5000 rpm for 30 s.

##### CVD Growth of Re‐ and V‐Doped TMDC Monolayers

All the CVD growths were conducted in a 2‐inch tube furnace. The temperature ramping rate was 30 °C min^−1^. Crucibles containing sulphur (99.999%, Fujifilm) and selenium (99.99%, Strem Chemicals) were kept at ≈180 °C and ≈300 °C during growth, respectively. To avoid possible contamination, each quartz tube was assigned for growing one specific Re‐ and V‐doped TMDCs. For Re‐ and V‐doped MoS_2_ monolayers, the growth was performed at 750 °C for 10 min with 200 sccm high‐purity argon as carrier gas. For Re‐ and V‐doped WS_2_ and WSe_2_ monolayers, the growth was performed at 800 °C for 10 min with 200 sccm high‐purity Ar/H_2_ (5%) forming gas.

##### Raman and PL

First, the Re‐ and V‐doped TMDCs grown on sapphire substrates were transferred onto SiO_2_ (285 nm)/Si substrates. Then, Raman and PL measurements were performed using a laser confocal microscope (Tokyo Instruments, Nanofinder FLEX). A 532 nm excitation laser with a spot size of 1 µm was focused onto the sample surface. The Raman/PL signals of samples were detected by an electron multiplying CCD detector through a grating with 2400 grooves mm^−1^ for Raman and 150 grooves mm^−1^ for PL, respectively.

##### STEM and EELS

STEM images were acquired by using JEOL 2100F microscope equipped with a JEOL‐DELTA correctors and the cold field emission gun operating at 60 kV. The probe current was about 25–30 pA. The convergence semiangle was 35 mrad and the inner acquisition semiangle was 79 mrad. The EELS core loss spectra were taken by using Gatan low‐voltage quantum spectrometer.

##### FET Fabrication and Measurements

The Re‐ and V‐doped TMDC monolayers were transferred onto SiO_2_ (285 nm)/Si substrates first. Then, a LED photolithography and oxygen plasma etching were conducted on Re‐ and V‐doped TMDC monolayers to define the channel shape. Next, another LED photolithography was repeated to pattern the electrodes. E‐beam evaporator was used to deposit Cr/Au (1/50 nm) as contact. A standard lift‐off process was employed by rinsing the substrates in acetone and IPA sequentially. All the transistors have the same channel size with length: ≈4 µm and width: ≈20 µm. Measurements were carried out in a high vacuum of ≈2 × 10^–4^ Pa. The backgate bias (*V*_gs_) was swept between −30 and 30 V with a step of 1 V. The source‐drain bias (*V*_ds_) is 1 V.

##### Fabrication of V‐Doped WSe_2_ Contacted WSe_2_‐FETs

First, parallel gaps were fabricated on the 10% $X_{VW}^{V}$‐WSe_2_ monolayers using LED photolithography (patterning) and etched with oxygen plasma sequentially. Then, the etched V‐doped WSe_2_ monolayers were transferred onto CVD‐gown WSe_2_ monolayers. Next, the second patterning and etching process were performed to define the channel area. Finally, photolithography was applied to pattern the electrodes and Au (50 nm) film was deposited using e‐beam evaporator (Figure S7, Supporting Information).

##### DFT Simulation

The DFT simulation was performed within the generalized gradient approximation of the Perdew, Burke, and Ernzernhof functional as implemented in the Vienna ab initio simulation package.^[^ ^47^, ^48^ ^]^ Electron‐ion interactions were described using projector‐augmented wave pseudopotentials.^[^ ^49^ ^]^ The effective Hubbard U value was set to 4.2 eV for the V 3d state.^[^ ^50^, ^51^, ^52^ ^]^ In the calculation of V‐doped WSe_2_/WSe_2_ and Au/WSe_2_ contacts, the Grimme's DFT‐D2 method was employed for vdW correction.^[^ ^53^ ^]^ A plane‐wave kinetic‐energy cutoff of 600 eV and a k‐spacing of 0.2 Å^–1^ in reciprocal space were used to ensure that the energy converged to better than 1 meV atom^−1^.

## Conflict of Interest

The authors declare no conflict of interest.

## Supporting information



Supporting Information

## Data Availability

Research data are not shared.
