# Supplementary material for: Tunable Doping of Rhenium and Vanadium into Transition Metal Dichalcogenides for Two‐Dimensional Electronics
Source: Adv Sci (Weinh). 2021 Apr 2;8(11):2004438. doi: 10.1002/advs.202004438 (PMC8188190; doi:10.1002/advs.202004438)
Supplement: Supplementary file 1 — Supporting Information [file ADVS-8-2004438-s001.pdf]

© 2021 Wiley-VCH GmbH

ADVANCED SCIENCE
Open Access

Supporting Information

for *Adv. Sci.*, DOI: 10.1002/advs.202004438

Tunable Doping of Rhenium and Vanadium into Transition Metal Dichalcogenides for Two-Dimensional Electronics

Shisheng Li, Jinhua Hong, Bo Gao, Yung-Chang Lin, Hong En Lim, Xueyi Lu, Jing Wu, Song Liu, Yoshitaka, Tateyama, Yoshiki Sakuma, Kazuhito Tsukagoshi, Kazu Suenaga and Takaaki Taniguchi**

© 2021 Wiley-VCH GmbH

Supporting Information

Tunable Doping of Rhenium and Vanadium into Transition Metal Dichalcogenides for Two-Dimensional Electronics

Shisheng Li, Jinhua Hong, Bo Gao, Yung-Chang Lin, Hong En Lim, Xueyi Lu, Jing Wu, Song Liu, Yoshitaka, Tateyama, Yoshiki Sakuma, Kazuhito Tsukagoshi, Kazu Suenaga and Takaaki Taniguchi**

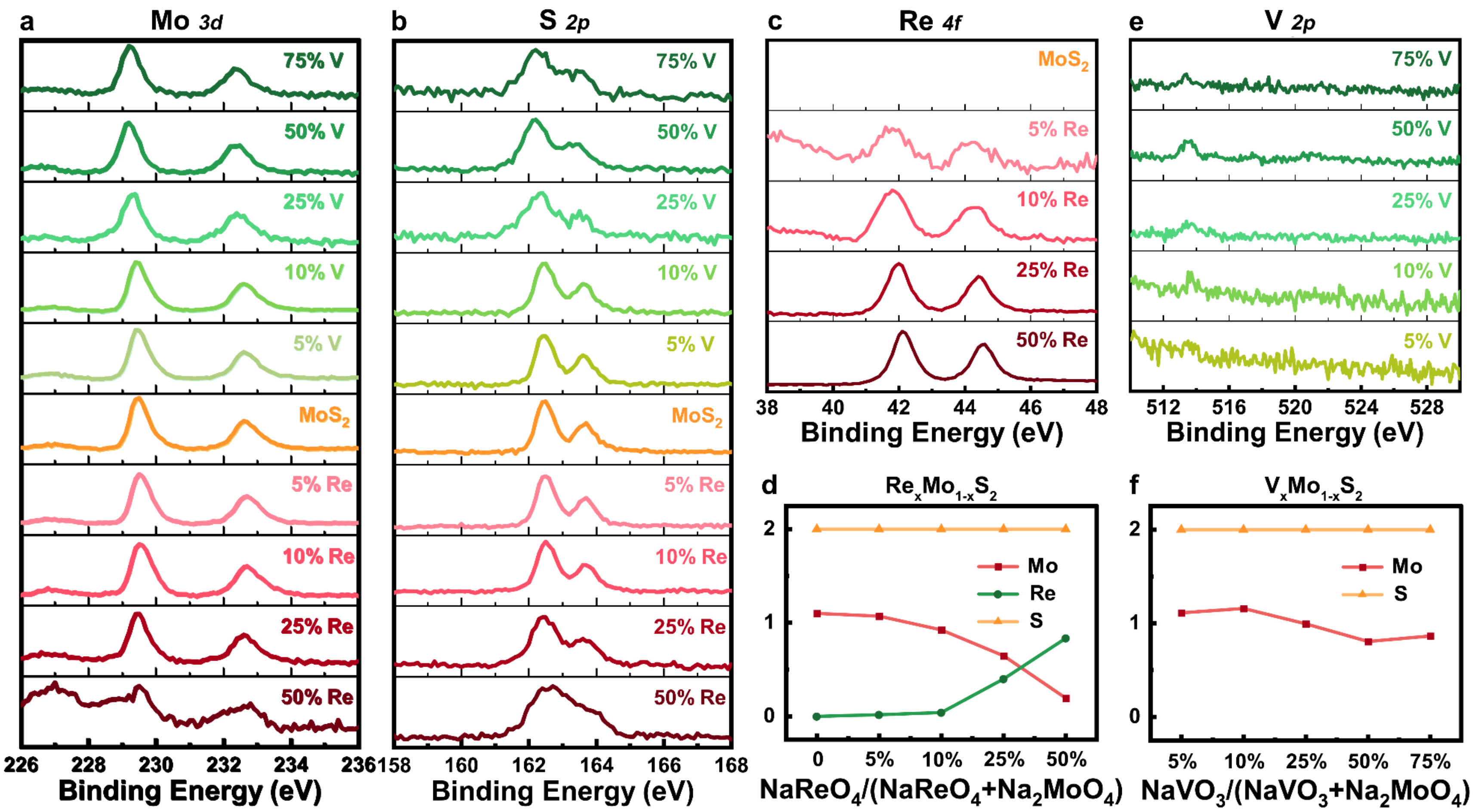

Figure S1. XPS spectra of Re and V-doped MoS_2 monolayers. XPS fine spectra of (a) Mo 3d, (b) S 2p, (c) Re 4f and (e) V 2p. (d) The evolution of Re and Mo atomic ratios in the as-grown Re-doped MoS_2 monolayers. (f) The evolution of Mo atomic ratios in the as-grown V-doped MoS_2 monolayers. S is calibrated to 2 in (d) and (f) for comparison. In figures a-c and e, y axis of the plots represent intensity with arbitrary unit.

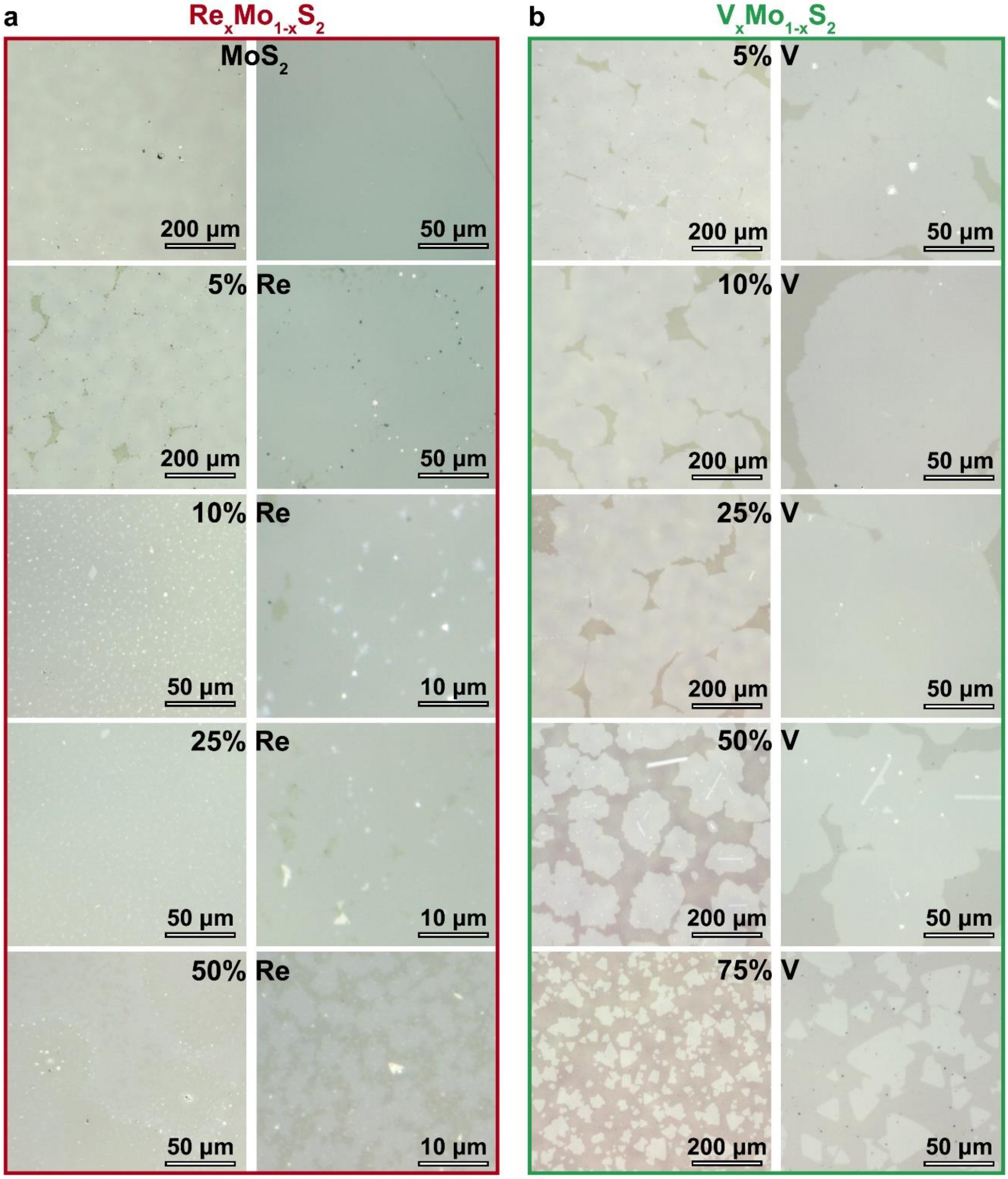

Figure S2. Optical images of Re and V-doped MoS_2 monolayers. (a) The ratios of $NaReO_4$ in mixed salt precursors, X_{ReMo}^{Re} are 0, 5%, 10%, 25% and 50%. (b) The ratios of $NaVO_3$ in mixed salt precursors, X_{VMo}^{V} are 5%, 10%, 25%, 50% and 75%.

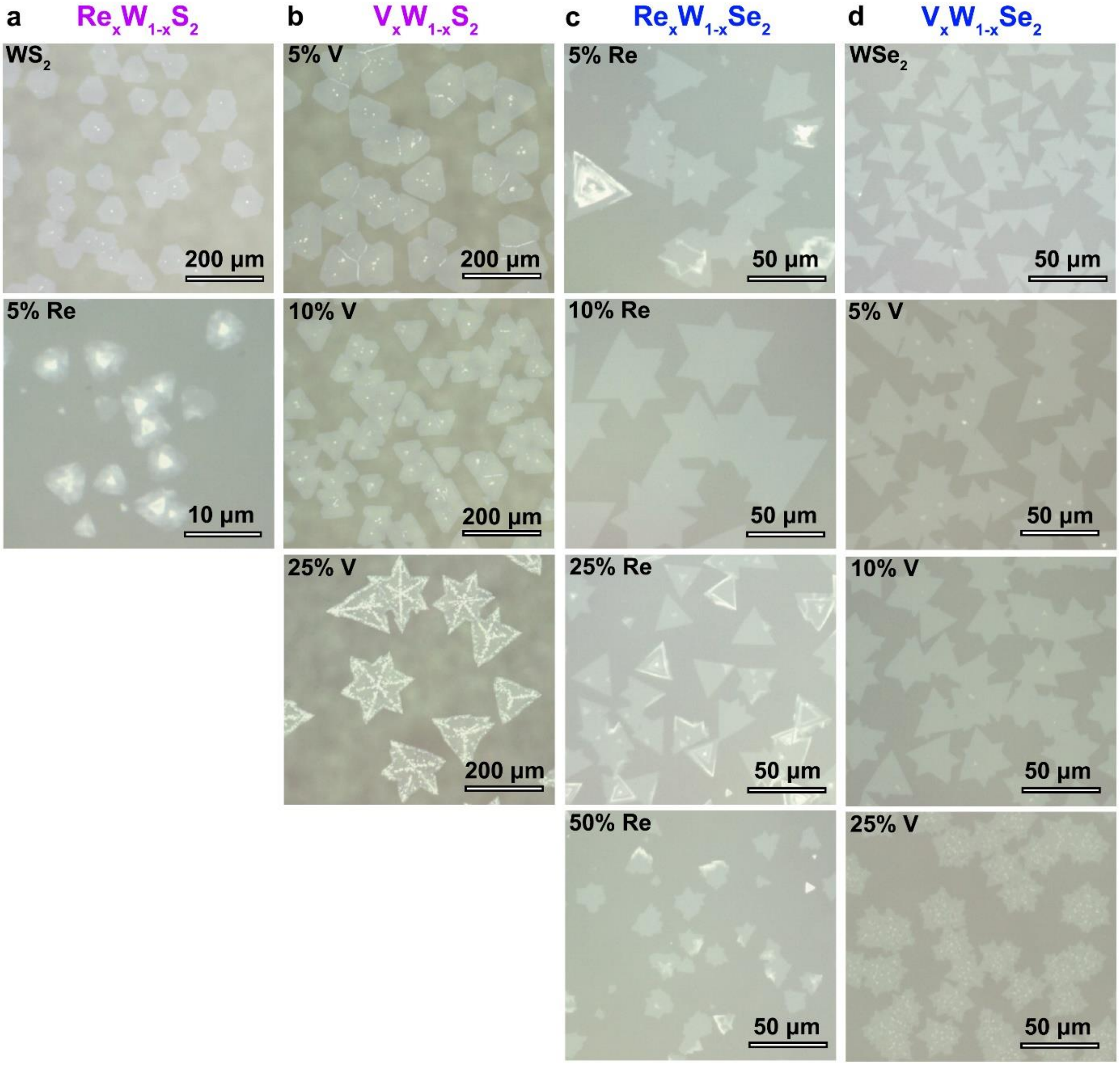

Figure S3. Optical images of Re and V-doped WS_2 and WSe_2. The ratios of dopant salts in mixed salt precursors: (a) X_{ReW}^{Re} are 0 and 5%. (b) X_{VW}^{V} are 5%, 10% and 25%; (c) X_{ReW}^{Re} are 5%, 10%, 25% and 50%; (d) X_{VW}^{V} are 0, 5%, 10% and 25%.

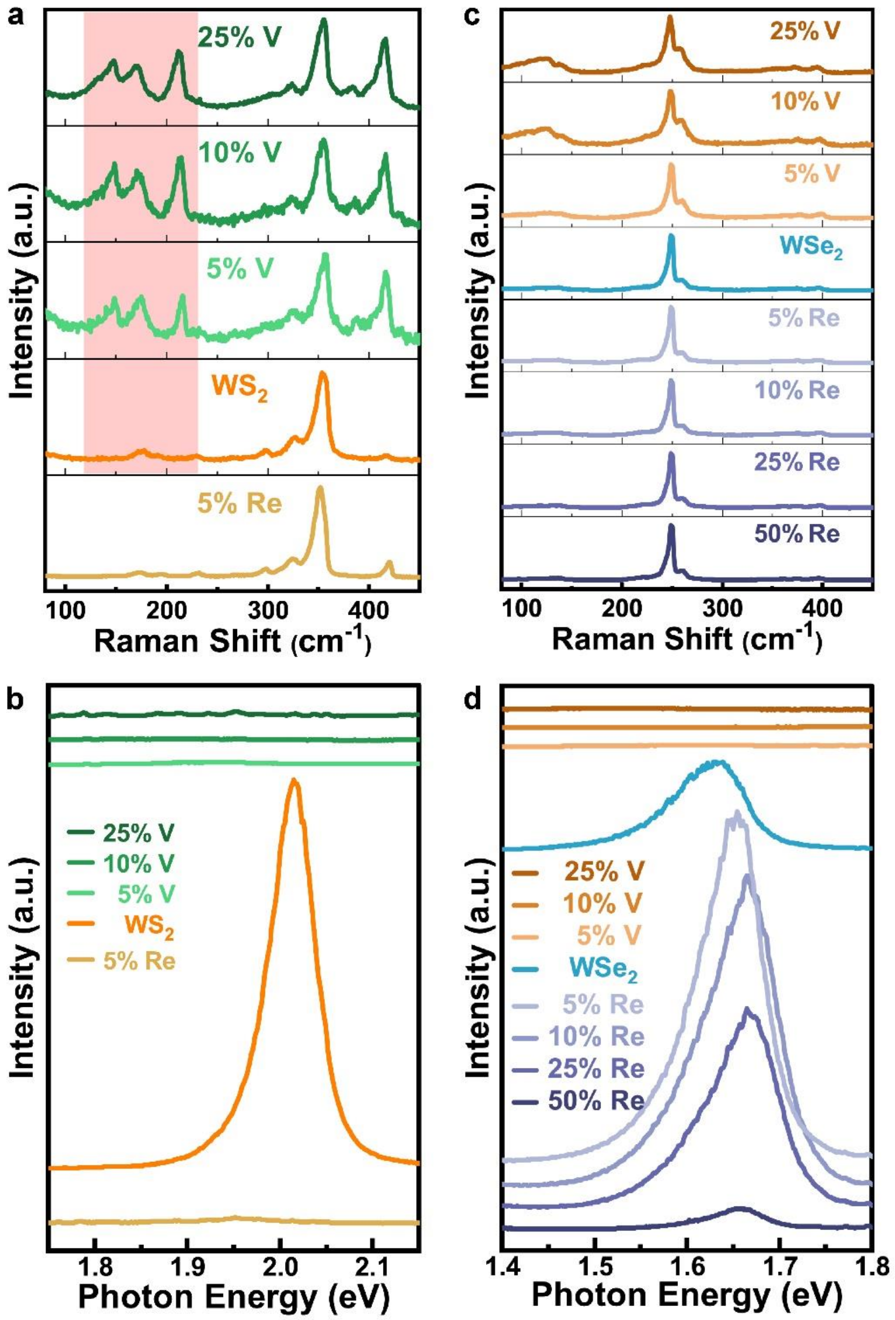

Figure S4. Raman and PL spectra of Re and V-doped WS_2 and WSe_2 monolayers. (a) Raman and (b) PL spectra of Re and V-doped WS_2 monolayers. (The 5% X_{ReW}^{Re}-WS_2 are multilayers). (c) Raman and (d) PL spectra of Re and V-doped WSe_2 monolayers.

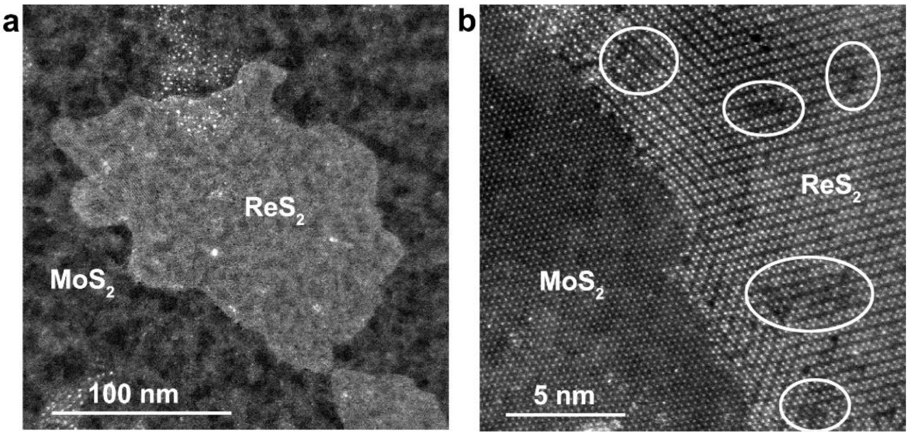

Figure S5. Structural characterization of 25% X^{Re}_{ReMo}-MoS$_2$ monolayers. (a) Low-magnification STEM image of a ReS_2 domain in MoS_2 matrix. (b) High-magnification STEM image showing the atomic structures of Re-doped MoS_2 and ReS_2. White circles indicate the substitutional Mo doping in the ReS_2 domain.

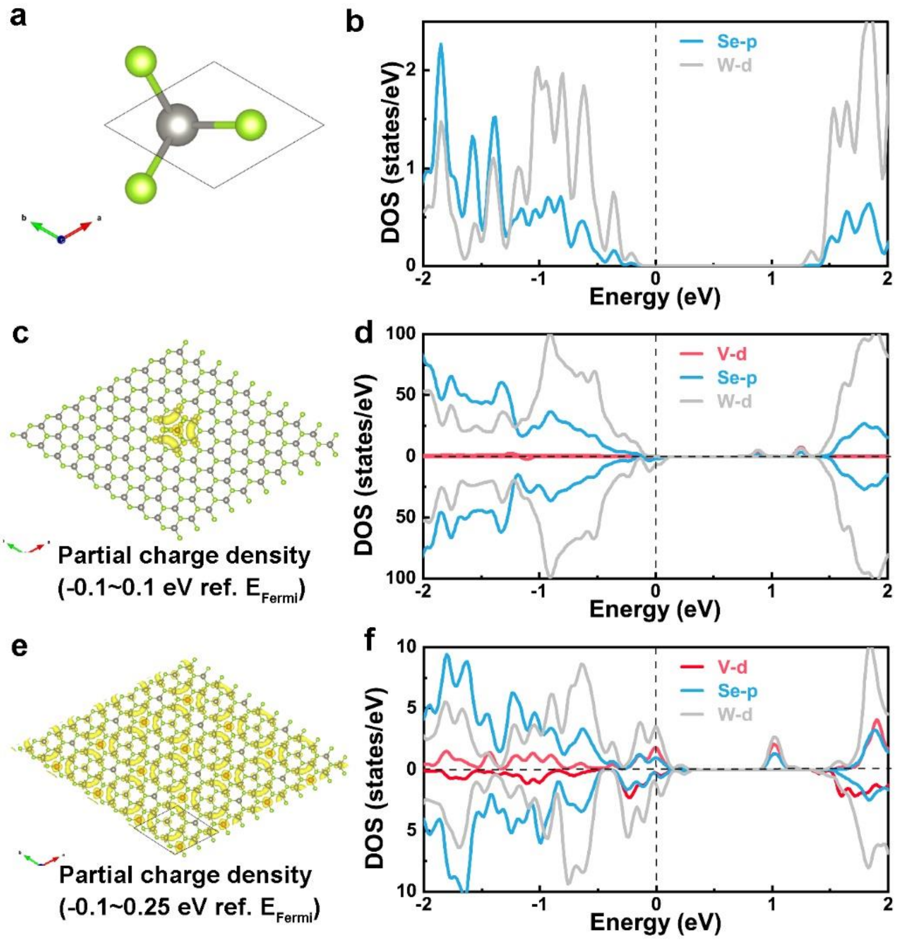

Figure S6. Charge densities and PDOSs of V-doped WSe_2 monolayers. (a) Unit cell and (b) PDOS of WSe_2 monolayer. (c) Partial charge density associated with the states from -0.1 to 0.1 eV relative to the Fermi level and (d) PDOS of 1 % V-doped WSe_2 monolayer. (e) Partial charge density associated with the states from -0.1 to 0.25 eV relative to the Fermi level and (f) PDOS of 11.1 % V-doped WSe_2 monolayer. In the PDOSs figures, the dashed lines indicate the Fermi level.

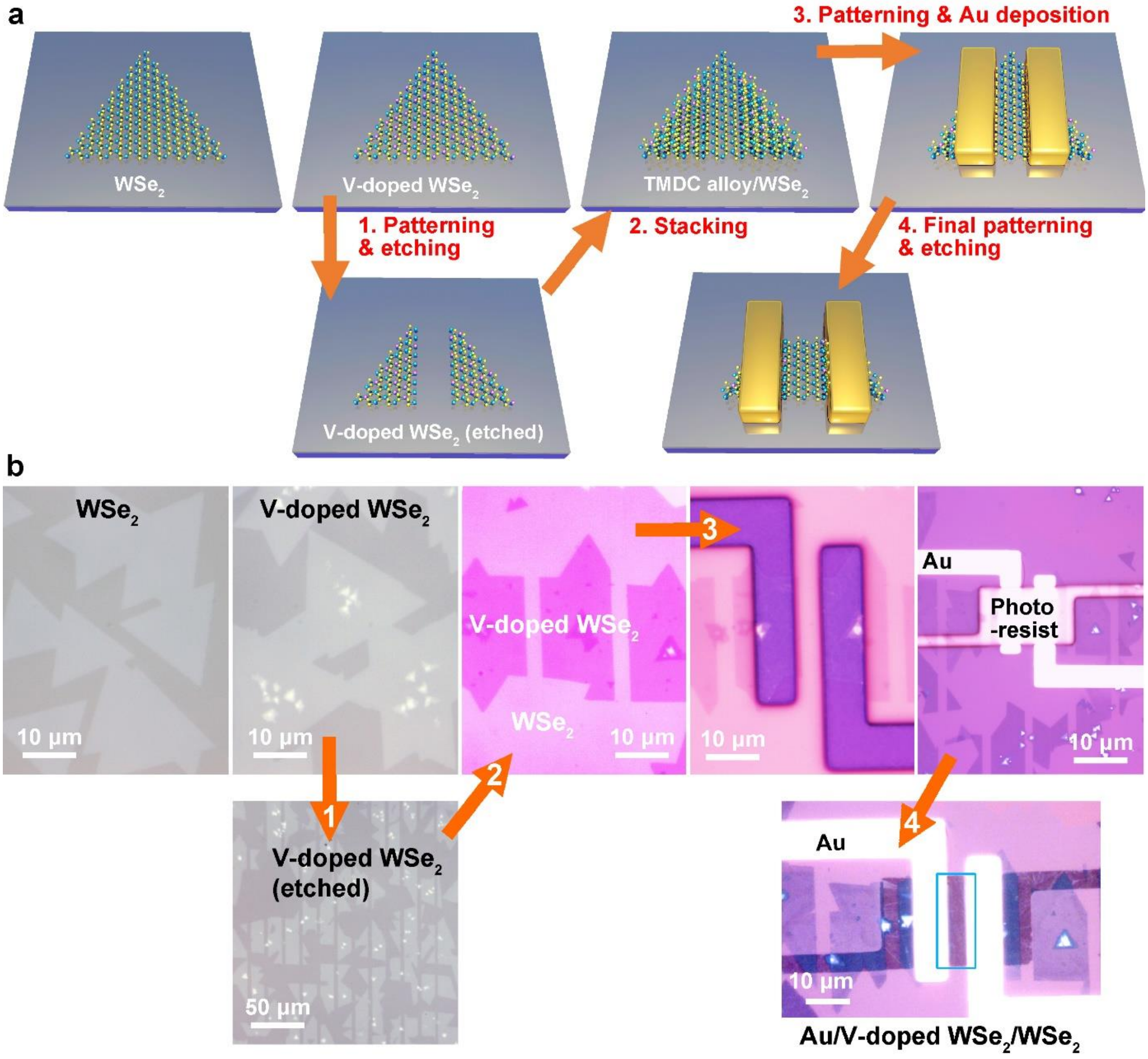

Figure S7. Fabrication of WSe$_2$-FETs with V-doped WSe$_2$ vdW contact. (a) Schematics and (b) corresponding optical images of the detailed fabrication process for 10% X_{VW}^{V}-WSe$_2$ monolayer contacted WSe$_2$-FETs.

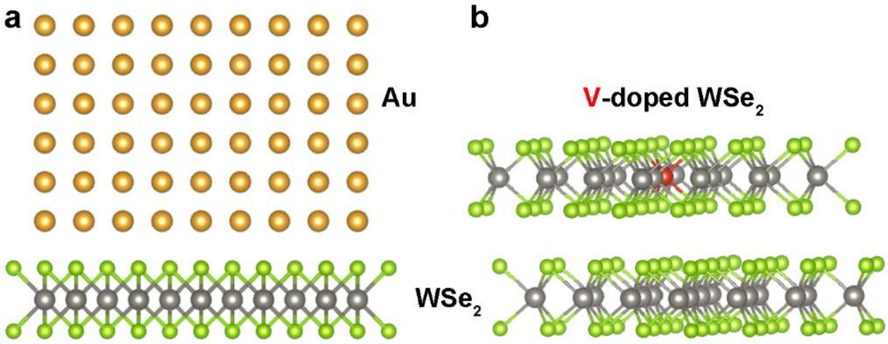

Figure S8. Two contact geometries in the DFT simulation. (a) Au/WSe_2 contact. (b) V-doped WSe_2/WSe_2 contact.
